# Experimental Models of Type-2 Diabetic Nephropathy

**DOI:** 10.1155/2012/218917

**Published:** 2012-03-19

**Authors:** Yasuhiko Tomino, Mark E. Cooper, Theodore W. Kurtz, Yoshio Shimizu

**Affiliations:** ^1^Division of Nephrology, Department of Internal Medicine, Juntendo University Faculty of Medicine, Tokyo 113-8421, Japan; ^2^JDRF Danielle Albert Memorial Centre for Diabetes Complications, Diabetes Division, Baker IDI Heart and Diabetes Institute, Melbourne, VIC 3004, Australia; ^3^Department of Laboratory Medicine, University of California, San Francisco, San Francisco, CA 94143, USA

Type 2 diabetic nephropathy is one of the major long-term microvascular complications occurring in nearly 40% of diabetic patients and also a major cause of end-stage kidney disease (ESKD) throughout the world. It is assumed that the number of type 2 diabetes and diabetic nephropathy patients is increasing and that more and more patients will experience progressive renal disease due to lack of effective treatments. The pathogenesis of type 2 diabetic nephropathy includes genetic, metabolic (hyperglycemic), and/or hemodynamic factors such as glomerular hypertension and associated renal hypertrophy. There are many progressive factors in patients with type 2 diabetic nephropathy, but few if any specific treatments for human diabetic nephropathy based on the mechanisms of disease initiation and progression have been clearly identified. Thus, it is important to investigate and determine pathogenesis (mechanisms of initiation and/or progression) and treatments using various experimental models of type 2 diabetic nephropathy.

This special issue contains 11 papers, based on studies of various animal models, cell cultures, and human samples.

In the paper entitled “*Dietary restriction ameliorates diabetic nephropathy through anti-inflammatory effects and regulation of the autophagy via restoration of Sirt1 in diabetic Wistar fatty (fa/fa) rats: a model of type 2 diabetes*,” M. Kitada et al. examined the renoprotective effects of dietary restriction (DR) in Wistar fatty (*fa/fa*) rats (WFRs). DR ameliorated renal abnormalities including inflammation in WFRs. The decrease in Sirt1 levels, increase in acetylated-NF-**κ**B, and impaired autophagy in WFRs were improved by DR. The authors concluded that DR exerted anti-inflammatory effects and improved the dysregulation of autophagy through the restoration of Sirt1 in the kidneys of WFRs, which resulted in the amelioration of renal injuries in type 2 diabetes.

In the paper entitled “*High glucose increases metallothionein expression in renal proximal tubular epithelial cells*,” D. Ogawa et al. found that the renal tissues in adult male diabetic rats induced by streptozotocin were stained with antibodies for MT-1/-2. MT-1/-2 expression was also evaluated in mProx24 cells, a mouse renal proximal tubular epithelial cell line, stimulated with high glucose medium and pretreated with the antioxidant vitamin E. These observations suggest that MT-1/-2 is induced in renal proximal tubular epithelial cells as an antioxidant to protect the kidney from oxidative stress and that it may offer a novel therapeutic target against diabetic nephropathy.

 In the paper entitled “*Targeted proteomics of isolated glomeruli from the kidneys of diabetic rats: sorbin and SH3 domain containing 2 is a novel protein associated with diabetic nephropathy*,” S. Nakatani et al. examined the protein expression in the isolated glomeruli from spontaneous type 2 diabetic (OLETF) rats and their age-matched control littermates (LETO) in the early and proteinuric stages of diabetic nephropathy using QSTAR Elite LC-MS/MS. Sorbin and SH3 domain containing 2 (SORBS2) was significantly upregulated in both stages of diabetic nephropathy. Immunohistochemical and quantitative PCR analyses revealed upregulation of SORBS2 in the podocytes of glomeruli of OLETF rats. These findings suggested that SORBS2 may be associated with the development of diabetic nephropathy possibility by reorganization of actin filaments.

In the paper entitled “*Role of T cells in type 2 diabetic nephropathy*,” C.-C. Wu et al. reviewed the current information concerning the role of T cells in the development and progression of type 2 diabetic nephropathy. Specific emphasis is placed on the potential interaction and contribution of the T cells to renal damage. The therapeutic strategies involving T cells in the treatment of type 2 diabetic nephropathy are also reviewed. Improving knowledge of the recognition of T cells as significant pathogenic mediators in diabetic nephropathy reinforces the possibility of finding new potential therapeutic targets that may be translated into future clinical treatments.

In the paper entitled “*Involvement of F-Actin in chaperonin-containing t-complex 1 beta regulating mouse mesangial cell functions in a glucose-induction cell model*,” J.-S. Chen et al. investigated the role of chaperonin-containing t-complex polypeptide 1 beta (CCT2) in the regulation of mouse mesangial cell (mMC) contraction, proliferation and migration with filamentous/globular- (F/G-) actin ratio under high glucose induction. Major results were as follows: (1) low CCT2 or high glucose showed the ability to attenuate F/G-actin ratio, (2) groups with low F/G-actin ratio all showed less cell contraction, and (3) suppression of CCT2 may reduce the proliferation and migration which were originally induced by high glucose. The authors concluded that CCT2 can be used as a specific regulator for mMC contraction, proliferation, and migration affected by glucose, in which the mechanism may involve the alteration of F-actin, particularly for cell contraction.

In the paper entitled “*Diabetic nephropathy amelioration by a low-dose sitagliptin in an animal model of type 2 diabetes (Zucker diabetic fatty rat)*,” C. Mega et al. performed studies to assess the effect of chronic low-dose sitagliptin, a dipeptidyl peptidase 4 inhibitor, on metabolic profile and on renal lesions in a rat model of type 2 diabetic nephropathy, the Zucker diabetic fatty (ZDF) rat. The authors concluded that chronic low-dose sitagliptin treatment was able to ameliorate diabetic nephropathy, which might represent a key step forward in the management of type 2 diabetes and this serious complication.

In the paper entitled “*Role of mindin in diabetic nephropathy*,” M. Murakoshi et al. suggested that certain inflammatory biomarkers should be useful for diagnosis or monitoring of diabetic nephropathy. Mindin (spondin 2) is a member of the mindin-/F-spondin family of secreted extracellular matrix (ECM) proteins. Recent studies have showed that mindin is essential for initiation of innate immune response and represents a unique pattern-recognition molecule in the ECM. The authors previously demonstrated that the levels of urinary mindin in patients with type 2 diabetes were higher than those in healthy individuals. Therefore, the authors suggest that urinary mindin may be a potent biomarker for the development of diabetic nephropathy.

In the paper entitled “*New experimental models of diabetic nephropathy in mice models of type 2 diabetes: Efforts to replicate human nephropathy*,” M. J. Soler et al. explained that the generation of new experimental models of diabetic nephropathy created by crossing, knockdown, or knockin of genes continues to provide improved tools for studying diabetic nephropathy. These models provide an opportunity to search for new mechanisms involving the development of diabetic nephropathy, but their shortcomings should be recognized as well. Moreover, it is important to recognize that the genetic background has a substantial effect on the susceptibility to diabetes and kidney disease development in the various models of diabetes.

In the paper entitled “*Osmolarity and glucose differentially regulate aldose reductase activity in cultured mouse podocytes*,” B. Lewko et al. examined whether aldose reductase (AR), the enzyme implicated in diabetic complications in different tissues, is modulated by high glucose and osmolarity in podocytes. Hyperosmolarity acutely stimulated AR expression and activity, with subsequent increase of AR expression but decrease of activity. High glucose also elevated AR protein level; however, this was not accompanied by respective enzyme activation. Furthermore, high glucose appeared to counteract the osmolarity-dependent activation of AR. The authors concluded that AR is modulated by high glucose and increased osmolarity in a different manner in podocytes. Posttranslational events may affect AR activity independent of enzyme protein amount. Activation of AR in podocytes may be implicated in diabetic podocytopathy.

In the paper entitled “*Signaling mechanisms in the regulation of renal matrix metabolism in diabetes*,” M. M. Mariappan reviewed that mTOR-(mammalian target of rapamycin-) regulated pathways are pivotal in orchestrating high-glucose-induced production of extracellular matrix (ECM) proteins leading to functional and structural changes in the kidney culminating in adverse outcomes. Understanding signaling pathways that influence individual matrix protein expression could lead to the development of new interventional strategies. This review will highlight some of the diverse components of the signaling network stimulated by hyperglycemia with an emphasis on extracellular matrix protein metabolism in the kidney in diabetes.

In the paper entitled “*An angiotensin II type 1 receptor blocker, prevents renal injury via inhibition of the Notch pathway in Ins2 Akita diabetic mice*,” M. Koshizaka et al. showed that telmisartan inhibited the angiotensin II-induced increased expression of transforming growth factor *β* and vascular endothelial growth factor A which could directly activate the Notch signaling pathway in cultured podocytes. The authors indicated that the telmisartan prevents diabetic nephropathy through the inhibition of the Notch pathway.

*Yasuhiko Tomino* *Yasuhiko Tomino* *Mark E. Cooper* *Mark E. Cooper* *Theodore W. Kurtz* *Theodore W. Kurtz* *Yoshio Shimizu* *Yoshio Shimizu*

